# Oropharyngeal Probiotic ENT-K12 Prevents Respiratory Tract Infections Among Frontline Medical Staff Fighting Against COVID-19: A Pilot Study

**DOI:** 10.3389/fbioe.2021.646184

**Published:** 2021-06-24

**Authors:** Qiang Wang, Xuan Lin, Xiaochen Xiang, Wanxin Liu, Ying Fang, Haiping Chen, Fang Tang, Hongyan Guo, Di Chen, Xiafen Hu, Qingming Wu, Baoli Zhu, Junbo Xia

**Affiliations:** ^1^Institute of Infection, Immunology and Tumor Microenvironment, Hubei Province Key Laboratory of Occupational Hazard Identification and Control, Medical College, Wuhan University of Science and Technology, Wuhan, China; ^2^Huarun WISCO General Hospital Affiliated to Wuhan University of Science and Technology, Wuhan, China; ^3^CAS Key Laboratory of Pathogenic Microbiology and Immunology, Institute of Microbiology, Chinese Academy of Sciences, Beijing, China; ^4^Savaid Medical School, University of Chinese Academy of Sciences, Beijing, China; ^5^Beijing Key Laboratory of Antimicrobial Resistance and Pathogen Genomics, Beijing, China; ^6^Department of Pathogenic Biology, School of Basic Medical Sciences, Southwest Medical University, Luzhou, China; ^7^Department of Pulmonary Medicine, Affiliated Hangzhou First People’s Hospital, Zhejiang University School of Medicine, Hangzhou, China

**Keywords:** oropharyngeal probiotic ENT-K12, respiratory tract infections, healthcare workers, COVID-19, group A β-hemolytic streptococcus

## Abstract

Healthcare workers at the frontline are facing a substantial risk of respiratory tract infection during the COVID-19 outbreak due to an extremely stressful work schedule and public health event. A well-established first-line defense on oropharyngeal microbiome could be a promising strategy to protect individuals from respiratory tract infections including COVID-19. The most thoroughly studied oropharyngeal probiotic product which creates a stable upper respiratory tract microbiota capable of preventing upper respiratory tract infections was chosen to evaluate the safety and efficacy on reducing episodes of upper respiratory tract infections for COVID-19 healthcare workers. To our knowledge to date, this is the very first study describing the beneficial effects of oropharyngeal probiotic been administered by healthcare workers during the COVID-19 pandemic. In this randomized controlled trial, we provided the probiotics to frontline medical staff who work in the hospitals in Wuhan and had been in close contact with hospitalized COVID-19 patients for prophylactic use on a daily basis. Our finding suggests that oropharyngeal probiotic administration significantly reduced the incidence of respiratory tract infections by 64.8%, reduced the time experiencing respiratory tract infections and oral ulcer symptoms by 78%, shortened the days absent from work by 95.5%, and reduced the time under medication where there is no record of antibiotic and anti-viral drug intake in the probiotic group. Furthermore, medical staff treated with Bactoblis experienced sustained protection from respiratory tract infections since the 10th day of oropharyngeal probiotic administration resulting in an extremely low incidence rate of respiratory tract infections.

## Introduction

Frontline medical staff fighting against COVID-19 are facing a substantial risk of respiratory tract infection during the COVID-19 outbreak due to an extremely stressful work schedule and public health event. During January 20, 2020 and February 5, 2020, it has been observed that the case infection rate of healthcare workers (2.10%) was dramatically higher than that of non-healthcare workers (0.43%) in a tertiary hospital during the early stage of COVID-19 outbreak in Wuhan (Lichun et al., 2020). Not only the COVID-19, but the general respiratory tract infection risk of healthcare workers is clearly higher than that of non-healthcare workers (Yaowen et al., 2016). Healthcare workers play an essential role in fighting the unexpected pandemic. Providing effective protection for healthcare workers from respiratory infections is essential.

Recent studies have shown that the microbiota in the lung contributes to immunological homeostasis and can potentially, when affected by dysbiosis, alter susceptibility to viral infection, and with respect to COVID-19, a highly significant difference in the lung microbiota composition has been observed between patients with COVID-19 pneumonia and healthy subjects, implying a dysbiosis occurred in the lung microbiota of patients who had a pathogen-enriched microbiota; not only can lung microbiota dysbiosis create a more fertile ground for viral aggression, but it can also promote a worsening of the patient condition (Zijie et al., 2020). A half-year prospective cohort study showed that nasopharyngeal microecological imbalance was caused by trans-colonization of oral microbiota, leading to upper respiratory tract infections (Lirong et al., 2020). An earlier study also demonstrated that inducing immune responses that were localized to the airway were more protective against challenge with pathogenic human coronaviruses, and that the strategy of inducing innate and specific immune responses at airway epithelium cells could also be useful in the context of protecting human host from other pathogenic respiratory viruses (Jincun et al., 2016).

Apparently, as in many other respiratory pathogens, the coronavirus is transmitted from an infected person’s mouth or nose through small liquid particles when they cough, sneeze, speak, or breathe heavily. Hence, the protection from these liquid particles of different sizes, ranging from larger “respiratory droplets” to smaller “aerosols,” become a key target for protection. Specifically, for healthcare workers who are in close contact with hospitalized COVID-19 patients, it is extremely important to make sure that the healthcare workers are fully equipped with proper protection, e.g., secondary and tertiary prevention. One possible additional way to protect human host from respiratory tract infections is to boost the immunity and maintain a healthy and balanced microflora at oropharyngeal environment of individuals via probiotics administration (Olga et al., 2014; Clark et al., 2016; Martens et al., 2018). Probiotics are live, non-pathogenic bacteria that may have an effect on both viral and bacterial infection. Recent trials have demonstrated that probiotics may impact the immune system, but only the commensal oral probiotic strain *Streptococcus thermophilus* ENT-K12 has been shown to successfully colonize the oral cavity and to modulate the nasopharyngeal microbiota (Ilchenko et al., 2019a, b). In fact, a slow-dissolved oropharyngeal probiotic formula containing ENT-K12 has been clinically demonstrated to improve the upper respiratory tract microbiota protecting the host from pathogenic bacteria, fungi, and viruses thereby reducing the incidence of viral respiratory tract infections and bacterial co-infections (Wilcox et al., 2019). With several clinical studies providing the safety and effectiveness (Wilcox et al., 2019), the said probiotic formula appears to be a promising agent to be administered for prophylactic or probiotic treatments to protect individuals during the outbreak of seasonal or emerging respiratory infection diseases.

## Materials and Methods

The trial was conducted according to the criteria set by the Declaration of Helsinki and with the approval of the local ethics committee, the Ethics Committee of Medical school of Wuhan University of Science and Technology (registration number 202003). All the medical staff who participated in the trial were informed of the trial methods and signed the consent. The study product, Bactoblis oropharyngeal probiotic formula, is formulated in the form of slowly dissolving oral lozenges by Probionet GmbH (Herisau, Switzerland); the preparation of this formula used in the clinical trial contained no less than 1 billion colony-forming units (cfu)/lozenge of S. *thermophilus* ENT-K12 over shelf-life.

The multicenter, open, randomized controlled clinical trial was conducted on 200 frontline medical staff enrolled in Wuhan, China, and treated between March 5, 2020 and April 5, 2020. All the enrolled individuals were healthy doctors and nurses 20–65 years of age and work in close contact with hospitalized COVID-19 patients; while taking care of the hospitalized COVID-19 patients, the subjects are fully equipped with proper protection, e.g., secondary and tertiary prevention. The following exclusion criteria were used: individuals who had an acute respiratory tract infection or have been diagnosed as COVID-19 infection, underwent antibiotic treatment at the time of enrolling, known allergy against milk proteins, and were immunocompromised or immunodeficient.

The enrolled medical staff were provided with a 1-month supply of study product and instructed to take 2 lozenges a day, taking a single lozenge after breakfast every morning and before bedtime after brushing their teeth every evening, respectively. The subjects were required to suck the lozenge until it is fully dissolved (approximately 4–5 min) and to make sure that the lozenge is not chewed or directly swallowed. They were instructed not to drink or swallow any substance for at least 1 h after the administration of the study product.

The study involved at least two visits over a 1-month period, screening visit (visit 1) and final visit (visit 2). Additional visits took place if the enrolled medical staff experienced symptoms of respiratory tract infections, so a diagnosis could be confirmed and if necessary, a prescription provided. At the screening visit (visit 1), the overall details of the study were explained to the enrolled medical staff and informed consent was obtained from the said individuals. The medical history of the individuals was reviewed and inclusion and exclusion criteria were confirmed. The medical staff was asked to maintain their standard diet and exercise routine.

The participated subjects were required to return for final visits after 30 days and return any unused study product. Compliance will be assessed by counting unused lozenges at the final visit; compliance criteria judged at ≥90% of dispensed lozenges consumed.

The subjects were instructed to contact the study physician at any time during the study in case of symptoms of respiratory tract infection or pneumonia, such as sore throat, fever more than 38°C, dyspnea, enlarged lymph nodes, and/or the appearance of abscesses (pus) or white patches on tonsils. At each visit, any adverse events were recorded.

### Objectives

The primary objective of this study is to investigate the benefits of oropharyngeal probiotic in preventing respiratory tract infections in frontline medical staff who are in close contact with COVID-19 hospitalized patients during the COVID-19 outbreak that causes an extremely stressful work schedule. The secondary objective is to investigate the incidence rate of COVID-19 in hospital pneumonia infection, resorting to antibiotic therapy, treatment with antipyretics, anti-viral drugs, and steroids, and working days lost during the episodes of respiratory infections. The onset of side effects while the product was being administered has also been observed.

### Statistical Analysis

SPSS version 25.0 software is used for statistical analysis. For baseline characteristics of participants, M ± SD was used to describe the variation degree of samples between groups, and M (P_25_–P_75_) was used to describe if the data did not follow normal distribution. For the quantitative data of normal distribution, the difference between populations was inferred by the two independent samples *t*-test (Wilcoxon’ s rank sum test was used for the non-normal distribution data), and χ^2^ test or Fisher’s exact test was used for the qualitative data. The Kaplan–Meier statistic was used to estimate the level of protection of respiratory tract infection by administration of oropharyngeal probiotic over time. Survival analysis was used to determine the difference in cumulative morbidity among patients under different conditions during clinical observation.

## Results

One-hundred medical staff were treated with two lozenges of study product daily for 30 consecutive days. The other 100 medical staff served as the control group in the same period. As shown in Table 1, the two groups did not differ in their baseline characteristics. Seven subjects dropped out of the study on the first few days after enrollment for personal reasons. Deducting the seven subjects dropping out from the study, 98 medical staff were treated for 30 days with probiotic, while 95 did not receive probiotic and were considered to be the control group. Compliance with the probiotic treatment was very good and well tolerated by the subjects with no side effects reported. The probiotic group consisted of 30 males and 68 females, mean age 36.13 ± 8.62 years. The control group consisted of 26 males and 69 females, mean age 35.74 ± 8.88 years. The two groups had no statistically significant differences in age and gender. As per the professional category of enrolled medical staff, the probiotic group consisted of 10 resident doctors, 20 attending doctors, 23 associate chief doctors, 7 chief doctors, and 38 nurses, with mean daily working hours 7.09 ± 1.17 and average 9 beds to attend. The control group consisted of 11 resident doctors, 17 attending doctors, 18 associate chief doctors, 3 chief doctors, and 46 nurses, with mean daily working hours 7.02 ± 1.09 and average 9 beds to attend. The two groups had no statistically significant differences in the constituent ratio of positions they hold, daily working hours, and beds to attend. Regarding the vaccination status of the enrolled medical staff, only 1 out of 98 in the probiotic group had pneumococcal vaccine and 4 out of 98 had influenza vaccine, while 1 out of 95 in the control group had pneumococcal vaccine and 4 out of 95 had influenza vaccine. The two groups had no statistically significant differences in the vaccination status.

**TABLE 1:** The baseline characteristics of participants.

	**Probiotic group**	**Control group**	***P* value**
Age	36.13 ± 8.62	35.74 ± 8.88	0.754^c^
Gender			0.620^b^
Male	30 (30/98)	26 (26/95)	
Female	68 (68/98)	69 (69/95)	
Professional category			0.522^b^
Resident Doctor	10	11	
Attending Doctor	20	17	
Associate Chief Doctor	23	18	
Chief Doctor	7	3	
Nurse	38	46	
Daily working hours	7.09 ± 1.17	7.02 ± 1.09	0.664^c^
Beds to attend	9 (7–15)	9 (6–12)	0.276^a^
Pneumococcal vaccine			0.982^b^
No	97	94	
Yes	1	1	
Influenza vaccine			0.964^b^
No	94	91	
Yes	4	4	

*X ± S is used to describe the average level and variability of the data.*

*^*a*^Wilcoxon’s rank sum test.*

*^*b*^Chi-square test.*

*^*c*^Student’s *t*-test.*

Table 2 shows the data on the prevalence of respiratory tract infection episodes during March 5, 2020 and April 5, 2020. Prophylaxis with oropharyngeal probiotic significantly reduced the incidence of respiratory tract infections by 64.8% (*p* < 0.005) comparing with the control group, of which 8 episodes of respiratory tract infections were observed in the group of 98 probiotic-treated medical staff and 22 episodes were observed in the group of 95 non-treated medical staff, with none of the episodes having been confirmed to be COVID-19 infection verified by SARS-CoV-2 nucleic acid test (data not shown). Key symptoms of the respiratory tract infections observed during this study include sore throat, cough and itchy throat, low fever, nasal congestion and dizziness, acute otitis media, and oral ulcer. Serological diagnosis including blood cytokine detection were not conducted due to the highly occupied workload for the frontline medical staff during the study period. However, the subjects having professional medical skills have been able to express and diagnose their symptoms accurately, hence, confirming the episodes of upper respiratory tract infections accurately. Among the eight episodes observed in the probiotic group, four showed symptoms of mainly sore throat, three showed symptoms of mainly cough and itchy throat, and one subject experienced low fever. As for the 22 episodes observed in the control group, 10 showed symptoms of mainly sore throat, 3 showed symptoms of mainly cough and itchy throat, 5 subjects experienced low fever, 2 subjects had nasal congestion and dizziness or headache, 1 subject reported acute otitis media along with sore throat, and 1 subject had oral ulcer. Comparing the key symptoms of upper respiratory tract infections, a trend of decreased incidence of key symptoms was observed during the prophylaxis with oropharyngeal probiotics, of which the incidence of sore throat was reduced by 61.3% and the incidence of low fever was reduced by 80.6% (*p* < 0.1). By comparison with the control group, the frontline medical staff in the probiotic group experienced a significantly lower number of days experiencing respiratory tract infection (RTi) symptoms (78%, *p* < 0.005). In fact, 23 days (0.23 days/person) of experiencing RTi symptoms were observed in the probiotic group whereas a total of 100 days (1.05 days/person) was observed in the control group. Meanwhile, treatment with oropharyngeal probiotic resulted in a significantly shorter (by 38%, *p* < 0.05) average duration of infection episodes (2.88 days/episode) compared with the control group (4.67 days/episode). Due to the reduction in total episodes, sick days, and duration of each episodes, the subjects treated with probiotic had significantly less days absent from work by 95.5% (*p* < 0.005); in other words, totally 3 days (0.03 days/person) of absence from work were reported in the probiotic group whereas totally 63 days (0.67 days/person) were reported in the control group.

**TABLE 2:** The difference analysis of each factor between two groups.

	**Probiotic group**	**Control group**	**Total**	***P* value**
Incidence of respiratory tract infections	8/98 (8.16%)	22/95 (23.16%)	30/193 (15.54%)	0.004^b^
Sore throat	4/98 (4.08%)	10/95 (10.53%)	14/193	0.084^b^
Cough/itchy throat	3/98 (3.06%)	3/95 (3.16%)	6/193	0.969^b^
Low fever	1/98 (1.02%)	5/95 (5.26%)	6/193	0.090^b^
Nasal congestion/dizziness	0	2/95 (2.11%)	2/193	0.149^b^
Acute otitis media	0	1/95 (1.05%)	1/193	0.492^c^
Oral ulcer	0	1/95 (1.05%)	1/193	0.492^c^
Sick days (days/person)	0.23 ± 0.961	1.05 ± 2.317	0.64 ± 1.807	0.004^a^
Duration of each episode (days/episode)	2.88 ± 2.031	4.67 ± 2.652	4.17 ± 2.592	0.025^a^
Days of absence from work (days/person)	0.03 ± 0.225	0.67 ± 2.322	0.35 ± 1.664	0.002^a^
Taking Chinese medicine (days/person)	0.16 ± 0.905	0.72 ± 1.939	0.44 ± 1.527	0.006^a^
Taking antibiotics (days/person)	0	0.54 ± 1.827	0.26 ± 1.306	0.001^a^
Taking anti-viral drug (days/person)	0	0.48 ± 1.884	0.24 ± 1.341	0.006^a^
Taking anti-inflammatory drug (days/person)	0	0.11 ± 0.778	0.05 ± 0.547	0.150^a^

*The quantitative variables in this table are non-normally distributed because there are plenty of 0 values that appeared in the raw data; the M ± SD is used to describe the average level and the degree of variability instead of using the form of median (P_25_–P_75_).*

*^*a*^Wilcoxon’s rank sum test.*

*^*b*^Chi-square test.*

*^*c*^Fisher’s exact test.*

During the study period, when there was evidence of a respiratory tract infection, the enrolled medical staff in both groups were asked to record the variety and duration of drug treatment, and continue taking probiotic throughout the study period, including in case of antibiotic treatment required. Our data reveal that the frontline medical staff in the probiotic group took significantly less medication compared with the control group. The number of days taking Chinese herbal medicine was observed to be reduced by 77.8% (*p* < 0.01); it is reported that totally 16 days (0.16 days/person) of medication history on Chinese herbal medicine was observed in the probiotic group compared with totally 68 days (0.72 days/person) observed in the control group. Further, during the randomized controlled trial, participants in the probiotic group had no record of antibiotic and anti-viral drug intake compared with 51 and 46 days of antibiotic and anti-viral drug intake in the control group (*p* = 0.001 and *p* < 0.01), respectively. No intake of steroid/anti-inflammatory drug was also observed in the probiotic group compared with 10 days of intake of steroid/anti-inflammatory drugs in the control group.

**FIGURE 1:**
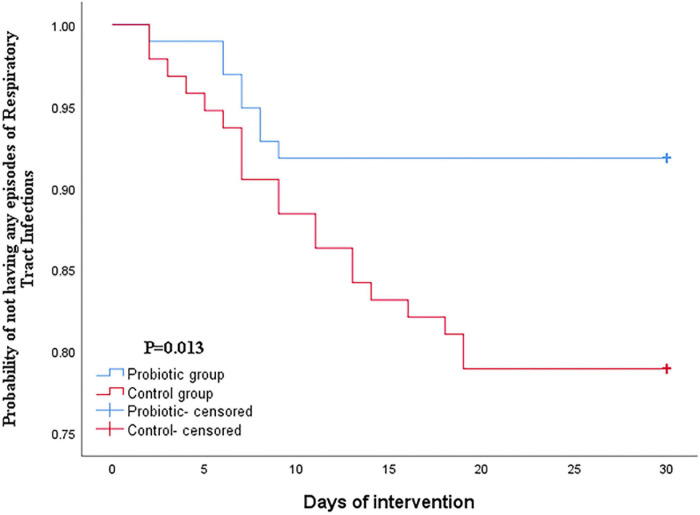
Kaplan–Meier curves of the cumulative incidence rates.

Furthermore, the Kaplan–Meier statistic was used to estimate the level of protection of respiratory tract infection by administration of oropharyngeal probiotic over time. As shown in Figure 1, the Kaplan–Meier curve of probability not having any episodes of respiratory tract infections decreased gradually from 1 on the first day of this trial. Notably, the cumulative incidence of respiratory tract infection stopped increasing on day 10 in the probiotic group. This implicates that frontline medical staff in the probiotic group experienced sustained protection from respiratory tract infections after a certain time of oropharyngeal probiotic administration resulting in an extremely lower incidence rate of respiratory tract infections comparing with control group (*p* = 0.013).

## Discussion

**FIGURE 2:**
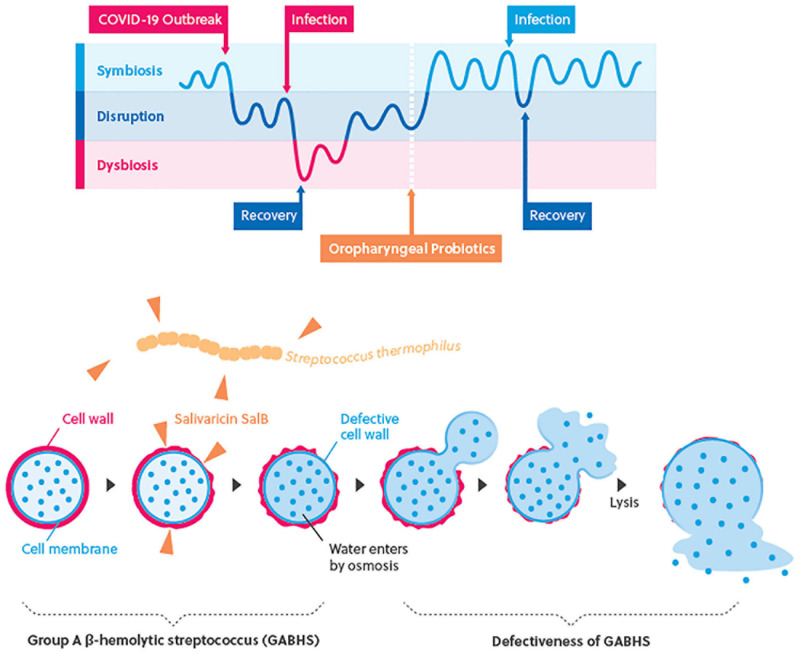
The hypothetical graph of interactions of host oropharyngeal microflora and immune responses.

Oral commensal microorganism has a central role in the homeostasis of airway mucosa and programming of the immune system (Harald et al., 2012). The said respiratory microbiota is sensitive to multiple factors, such as lifestyle, aging, environment, and disease (Mikari et al., 2018; Hongcheng et al., 2020). A cohort study has demonstrated that nasopharyngeal microecological imbalance was caused by trans-colonization of oral microbiota, leading to upper respiratory tract infections (Wing et al., 2019). Longitudinal observations have also found that psychological stress, mood states, or life events are associated with susceptibility of viral and intracellular bacterial infections and reduced lung function due to decreased cellular immune processes, such as those initiated by NK cells (Trueba and Ritz, 2012). Besides, SARS-CoV-2 mainly infect human angiotensin-converting enzyme 2 (ACE2), which is mainly expressed in tongue epithelial cells, while the viral load of SARS-CoV-2 in posterior oropharyngeal saliva samples was highest during the first week of symptom onset (Lirong et al., 2020), which further reveals that homeostasis of oropharyngeal mucosa that has an impact on the programming of the innate immune system could play an important role as a frontline defense and protect human host from respiratory tract infections including SARS-CoV-2. The results of this study indicate that oropharyngeal probiotic formula containing S. *thermophilus* ENT-K12 can reduce susceptibility to respiratory tract infections for frontline medical staff fighting against COVID-19. The mechanisms that underlie these effects have been described in previous studies, which includes colonization of the probiotics in oropharynx having the ability to locally release the two antibiotics, salivaricin A2 and B, to reduce the risk of colonization by group A β-hemolytic streptococcus including S. *pyogenes*; a common pathogen persists in the pharynx in a carrier state in approximately 10% of the population, which is a common cause of pharyngeal infections and a common bacterial pathogen that causes co-infection during viral infection (Upton et al., 2001). The salivaricin-producing probiotic strains has been proven to be of great value in the development of new and novel antibacterial therapies in this era of emerging antibiotic resistance via curing multi-resistant infections or reshaping the endogenous microbiota for prophylaxis purposes (Piewngam et al., 2018; Khan et al., 2019; Pascal et al., 2019; Barbour et al., 2020). As shown in Figure 2, the hypothetical graph indicates the host oropharyngeal microflora interactions which are divided into three states, symbiosis, disruption, and dysbiosis; when the host oropharyngeal microflora remains in the symbiosis state, the host is relatively more tolerant to the infections or environment factors which may disrupt the host’s immune responses. When frontline medical staff fighting COVID-19 switched to an unregulated and stressed work schedule during the pandemic outbreak, the disruption of symbiosis state could be induced and made the individuals more susceptible to the infections which further altered the host oropharyngeal microflora to become a dysbiosis state. Administration of oropharyngeal probiotics was able to establish a more balanced homeostatic relationship between the oropharyngeal microflora and the cells of the immune system responding to the infections and surrounding environment that shapes the oropharyngeal microflora, the said symbiosis state may provide more protections for the frontline medical staff fighting against COVID-19 from respiratory tract infections including COVID-19. The salivaricin, SalB, produced by oropharyngeal probiotics further protects the frontline medical staff from bacterial coinfections via disabling the reproduction of pathogenic bacteria on the oropharyngeal mucus surfaces (Barbour et al., 2016).

These results can be supported by our findings that prophylaxis with oropharyngeal probiotic formula containing ENT-K12 strain among COVID-19 frontline medical staff reduced the prevalence of respiratory tract infections by approximately 65%, sick days by approximately 80%, and no record of antibiotic, anti-viral drug and steroid/anti-inflammatory drug intake during this trial.

The proposed anti-viral capability of oropharyngeal probiotic has been attributed to the observed development of an innate immune response as revealed by detection of enhanced sufficient amount of IFN-γ in human saliva 10 h after oral lozenge administration (Di Pierro, 2020).

The administration of the ENT-K12 dose used in the present study has been shown to result in successful colonization of the oral cavity (Burton et al., 2010). Further, both the antibacterial as well as the anti-viral activity demonstrated in previous studies with the oropharyngeal probiotic may have contributed to the observed clinical benefits in the present study and may explain both the reduced incidence and duration of respiratory infections in the probiotic group of frontline medical staff.

According to our knowledge to date, this is the very first study describing the beneficial effects of oropharyngeal probiotic strain that has been administered by medical staff during an emerging disease outbreak. The Kaplan–Meier curve indicates that frontline medical staff treated with oropharyngeal probiotic experienced an extremely low incidence rate of respiratory tract infections after only 10 days of probiotic administration, suggesting that a healthier and more balanced oropharynx microflora homeostasis can be achieved by probiotic administration of 2 lozenges a day for about 10 days; this finding suggested that the colonization of ENT-K12 strain can be implemented by taking slow-dissolved probiotic formula 2 lozenges per day over about 10 days, which can be supported by a previous study demonstrating that K12 strain colonization can be achieved over 14 days while taking 1 lozenge per day or 3 days while taking 4 lozenges per day, defined by the sufficient amount of salivaricin A2 and B that had been detected in the saliva or on site, and the target habitat sites are pharynx, tongue, and buccal membranes which are also the habitats of agents causing pharyngitis (Horz et al., 2007). Besides the action mode of salivaricins described previously, a well-maintained oropharynx homeostasis status also helps keeping the human host tolerant of respiratory tract infections. Previous studies have demonstrated that the use of oropharyngeal probiotic as part of the complex therapy of recurrent tonsillitis for 30 days was characterized by a rapid relief of both local and general manifestations of recurrent tonsillitis, as well as a significant improvement in the microflora of the upper respiratory tract (Ilchenko et al., 2020; Kramarev et al., 2020). It is noted that in the control group, the incidence rate of respiratory tract infections increased linearly until the 20th day and seemed to slow down in the last 10 days during this trial, which may be explained by the fact that the number of hospitalized COVID-19 patients gradually decreased after they were cured and left the hospitals and the relief of COVID-19 pandemic in Wuhan area, thus relieved the shortage of medical resources during the emergence of the outbreak which caused extremely stressful work schedule and which also made the medical staff experience a lower immunity during the period of medical resource shortage.

Considering that the clinical benefit of oropharyngeal probiotic plays a role in creating a stable upper respiratory tract microbiota capable of protecting the host from respiratory tract infections, and its anti-viral capability to build a well-established first-line defense on the upper respiratory tract and oropharyngeal microbiome to protect individuals from respiratory tract infection could be a promising strategy to prevent respiratory tract infections, including COVID-19 infections.

Another public health crisis is that the number of pathogenic bacterial strains resistant to antibiotics has dramatically increased during the past decades so that some pathogenic microbes are totally insensitive to current antibiotics (De Francesco et al., 2010; Jakobsson et al., 2010; Beata and Anna, 2015); the new trends of research on antibacterial peptides produced by probiotics known as bacteriocins could provide beneficial features to substitute antibiotics or reduce the emergence of resistant strains. Fortunately, many human commensal bacterial species are able to be mobilized to produce bacteriocins and prevent bacterial infection at the external surface of human epithelia, which gives a great opportunity to cure multi-resistant infections or finely reshape the endogenous microbiota for prophylaxis purposes (Cotter et al., 2013; Sarah et al., 2019). According to the finding of this study and previous studies conducted with the same oropharyngeal probiotic formula, demonstrating a significant reduction by more than 90% of antibiotic use among subjects taking probiotics, administration of the slow-dissolved oropharyngeal probiotic could be a promising strategy to reduce the worldwide public health crisis related to the development of pathogenic antibiotic-resistant strains that caused respiratory tract infections, especially those that cause recurrent respiratory tract infections such as *Streptococcus pneumoniae*.

## Conclusion

This study evaluated the oropharyngeal probiotic in frontline medical staff in Wuhan fighting against COVID-19, and the results provide further support that administration of oropharyngeal probiotic which creates a stable upper respiratory tract microbiota can at least, for a short period of 20 days, protect frontline medical staff from upper respiratory tract infections, capable of reducing the duration of sick days, days absent from work, and days taking antibiotics and anti-viral drugs. This study certainly exhibits some limits: the absence of blind conditions, the small size of the sample which is not sufficient enough to detect the prevention effect of COVID-19 infections, not able to be involved in checking viral or streptococcal infection during the extremely busy work schedule, an inability to follow up the enrolled medical staff in the next subsequent month to assess further trends in infective oropharyngeal events due to that the number of hospitalized COVID-19 patients on the study sites gradually decreased when they are cured and leave hospital after April and May 2020, and an inability to analyze the composition of oropharyngeal microflora before and after oropharyngeal probiotic treatment, thus not able to directly prove the better homeostasis after a certain time of probiotic administration. Nevertheless, the findings of this study together with the beneficial clinical effects and improved oropharyngeal microbiota discovered from previous human clinical studies, and excellent tolerability and compliance, as well as the absence of side effects, demonstrated that the slow-dissolved oropharyngeal probiotic formula can be a valid solution in the prevention of respiratory tract infections.

## Data Availability Statement

The data analyzed in this study is subject to the following licenses/restrictions: The datasets used and/or analyzed during the current study are available from the corresponding author on reasonable request. Requests to access these datasets should be directed to QWa, wangqiang@wust.edu.cn.

## Ethics Statement

The studies involving human participants were reviewed and approved by Ethics Committee of Medical school of Wuhan University of Science and Technology (registration number 202003). The patients/participants provided their written informed consent to participate in this study.

## Author Contributions

BZ and QWu guided and completed the whole experimental design. FT, HG, and DC involved in the data collection. JX and XH were responsible for the arrangement of data. XX and WL were responsible for analyzing the data. YF and HC participated in the interpretation of the results. QWa and XL wrote the initial draft with all authors providing critical feedback and edits to subsequent revisions. QWa, JX, BZ, and QWu reviewed and revised the manuscript before submission, they are co-corresponding authors.

## Conflict of Interest

The authors declare that the research was conducted in the absence of any commercial or financial relationships that could be construed as a potential conflict of interest.
